# Supplementary material for: Prevalence of multidrug-resistant tuberculosis in East Africa: A systematic review and meta-analysis
Source: PLoS One. 2022 Jun 30;17(6):e0270272. doi: 10.1371/journal.pone.0270272 (PMC9246177; doi:10.1371/journal.pone.0270272)
Supplement: S2 Table — (DOCX) [file pone.0270272.s002.docx]

S2 Table. General characteristics of studies included in this meta-analysis in East Africa, 2007 to 2019

| Author (s)/References | Study area | Number of individuals screened for new cases of MDR-TB | Number of positive for New cases of MDR-TB | Number of Previously treated MDR-TB | Number of previously  treated MDR-TB who are positive |
| --- | --- | --- | --- | --- | --- |
| Admassu,2011 [[23](#_ENREF_23)] | Ethiopia | 410 | 19 | 150 | 74 |
| Mekonnen et al., 2015 [[24](#_ENREF_24)] | Ethiopia | 124 | 2 | 124 | 5 |
| Brhane et al.,2017 [[25](#_ENREF_25)] | Ethiopia | 105 | 3 | 31 | 7 |
| Biresaw et al., 2018 [[26](#_ENREF_26)] | Ethiopia | 381 | - | 381 | 7 |
| Girum et al., 2018 [[27](#_ENREF_27)] | Ethiopia | 7461 | 163 | 7461 | 1572 |
| Kerubo et al., 2016 [[28](#_ENREF_28)] | Kenya | 160 | 7 | 24 | 2 |
| Huerga et al., 2017 [[29](#_ENREF_29)] | Kenya | 169 | 4 | 169 | 24 |
| Umubyeyi et al., 2007 [[30](#_ENREF_30)] | Rwanda | 616 | 24 | 85 | 8 |
| Eldin et al., 2011. [[31](#_ENREF_31)] | Sudan | 43 | 8 | 71 | 17 |
| Sabeel et al., 2017[[32](#_ENREF_32)] | Sudan | 75 | 11 | 15 | 4 |
| Eldirdery et al., 2017[[33](#_ENREF_33)] | Sudan | 88 | 4 | 21 | 7 |
| Chonde et al., 2010 [[34](#_ENREF_34)] | Tanzania | 1019 | 11 | 148 | 40 |
| Range et al., 2012[[35](#_ENREF_35)] | Tanzania | 503 | 9 | 503 | 3 |
| Lukoye et al., 2011 [[36](#_ENREF_36)] | Uganda | 533 | 5 | 60 | 7 |
| Lukoye, 2013 [[3](#_ENREF_3)] | Uganda | 1397 | 178 | 140 | 35 |
| Okethwangu et al., 2019 [[37](#_ENREF_37)] | Uganda | 33 | 3 | 33 | 30 |
| Total | | 11720 | 451 | 9416 | 1842 |
