# Supplementary material for: Prevalence of multidrug-resistant tuberculosis in East Africa: A systematic review and meta-analysis
Source: PLoS One. 2022 Jun 30;17(6):e0270272. doi: 10.1371/journal.pone.0270272 (PMC9246177; doi:10.1371/journal.pone.0270272)
Supplement: S1 File — (DOCX) [file pone.0270272.s003.docx]

References of 16 studies used in meta-analysis

26. Demile B, Zenebu A, Shewaye H, Xia S, Guadie A: **Risk factors associated with multidrug-resistant tuberculosis (MDR-TB) in a tertiary armed force referral and teaching hospital, Ethiopia**. *BMC infectious diseases* 2018, **18**(1):1-10.

23. Admassu DMA: **Multidrug and Heteroresistant Mycobacterium**. *tuberculosis (comnent)* 2011, **378**:204.

24. Mekonnen F, Tessema B, Moges F, Gelaw A, Eshetie S, Kumera G: **Multidrug resistant tuberculosis: prevalence and risk factors in districts of metema and west armachiho, Northwest Ethiopia**. *BMC infectious diseases* 2015, **15**(1):1-6.

25. Brhane M, Kebede A, Petros Y: **Molecular detection of multidrug-resistant tuberculosis among smear-positive pulmonary tuberculosis patients in Jigjiga town, Ethiopia**. *Infection and drug resistance* 2017, **10**:75.

26. Demile B, Zenebu A, Shewaye H, Xia S, Guadie A: **Risk factors associated with multidrug-resistant tuberculosis (MDR-TB) in a tertiary armed force referral and teaching hospital, Ethiopia**. *BMC infectious diseases* 2018, **18**(1):1-10.

27. Girum T, Muktar E, Lentiro K, Wondiye H, Shewangizaw M: **Epidemiology of multidrug-resistant tuberculosis (MDR-TB) in Ethiopia: a systematic review and meta-analysis of the prevalence, determinants and treatment outcome**. *Tropical diseases, travel medicine and vaccines* 2018, **4**(1):1-12.

28. Kerubo G, Amukoye E, Niemann S, Kariuki S: **Drug susceptibility profiles of pulmonary Mycobacterium tuberculosis isolates from patients in informal urban settlements in Nairobi, Kenya**. *BMC infectious diseases* 2016, **16**(1):1-7.

29. Huerga H, Bastard M, Kamene M, Wanjala S, Arnold A, Oucho N, Chikwanha I, Varaine F: **Outcomes from the first multidrug-resistant tuberculosis programme in Kenya**. *The International Journal of Tuberculosis and Lung Disease* 2017, **21**(3):314-319.

30. Umubyeyi AN, Vandebriel G, Gasana M, Basinga P, Zawadi J, Gatabazi J, Pauwels P, Nzabintwali F, Nyiramasarabwe L, Fissette K: **Results of a national survey on drug resistance among pulmonary tuberculosis patients in Rwanda**. *The International Journal of Tuberculosis and Lung Disease* 2007, **11**(2):189-194.

31. Eldin GSS, Fadl-Elmula I, Ali MS, Ali AB, Salih ALG, Mallard K, Bottomley C, McNerney R: **Tuberculosis in Sudan: a study of Mycobacterium tuberculosis strain genotype and susceptibility to anti-tuberculosis drugs**. *BMC infectious diseases* 2011, **11**(1):1-8.

32. Sabeel S, Salih MA, Ali M, El-Zaki S-E, Abuzeid N, Elgadi ZAM, Altayb HN, Elegail A, Ibrahim NY, Elamin BK: **Phenotypic and genotypic analysis of multidrug-resistant Mycobacterium tuberculosis isolates from Sudanese patients**. *Tuberculosis research and treatment* 2017, **2017**.

33. Eldirdery MM, Intisar E, Mona O, Fatima A, Asrar M, Nuha Y: **Prevalence of multidrug-resistant tuberculosis among smear positive pulmonary tuberculosis patients in eastern Sudan**. *Am J Microbiol Res* 2017, **5**:32-36.

34. Chonde T, Basra D, Mfinanga S, Range N, Lwilla F, Shirima R, Van Deun A, Zignol M, Cobelens F, Egwaga S: **National anti-tuberculosis drug resistance study in Tanzania**. *The International Journal of Tuberculosis and Lung Disease* 2010, **14**(8):967-972.

35. Range N, Friis H, Mfaume S, Magnussen P, Changalucha J, Kilale A, Mugomela A, Andersen AB: **Anti-tuberculosis drug resistance pattern among pulmonary tuberculosis patients with or without HIV infection in Mwanza, Tanzania**. *Tanzania Journal of Health Research* 2012, **14**(4).

36. Lukoye D, Cobelens FG, Ezati N, Kirimunda S, Adatu FE, Lule JK, Nuwaha F, Joloba ML: **Rates of anti-tuberculosis drug resistance in Kampala-Uganda are low and not associated with HIV infection**. *PloS one* 2011, **6**(1):e16130.

37. Okethwangu D, Birungi D, Biribawa C, Kwesiga B, Turyahabwe S, Ario AR, Zhu B-P: **Multidrug-resistant tuberculosis outbreak associated with poor treatment adherence and delayed treatment: Arua District, Uganda, 2013–2017**. *BMC infectious diseases* 2019, **19**(1):1-10.
