# Supplementary material for: Prevalence of multidrug-resistant tuberculosis in East Africa: A systematic review and meta-analysis
Source: PLoS One. 2022 Jun 30;17(6):e0270272. doi: 10.1371/journal.pone.0270272 (PMC9246177; doi:10.1371/journal.pone.0270272)
Supplement: Attachment — Submitted filename: Few omments on- Prevalence of multidrug-resistant tuberculosis in East Africa paper Feb 2022.docx [file pone.0270272.s004.docx]

Finding

Comments

1. The paper needs revision in view of spelling, English grammar.

2. Plagiarism was found which was more than expected

3. Key word, prevalence ‘P’ letter was written in a lower letter

4. Since the topic of this manuscript focuses on Prevalence MDR in East African countries, the study area was not included countries fairly in the study, e.g. Djibouti, Somalia, Eritrea, and the rest and also the included countries not equally or fairly distributed the study area according to table 1 page 8 and line 202 e. g Ethiopia 5 times whereas Rwanda is one time and so on
