# Supplementary material for: Prevalence of multidrug-resistant tuberculosis in East Africa: A systematic review and meta-analysis
Source: PLoS One. 2022 Jun 30;17(6):e0270272. doi: 10.1371/journal.pone.0270272 (PMC9246177; doi:10.1371/journal.pone.0270272)
Supplement: Attachment — Submitted filename: Response to Reviewers.docx [file pone.0270272.s005.docx]

**Rebuttal Letter**

We would like to thank the Editor and reviewers for their valuable and generous comments on our manuscripts by devoting their time. We would like to appreciate the reviewer for the comments have given definitely help us to improve our manuscript. We have tried to edit the manuscript to address their concerns on the title of “**Prevalence of multidrug-resistant tuberculosis in East Africa: A systematic review and meta-analysis”** with ID of PONE-D-22-00221**.** Important corrected mistakes are highlighted with yellow color in the corrected manuscript.

**Editor comment**

1. Please ensure that your manuscript meets PLOS ONE's style requirements, including those for file naming.

**Authors’ response**

- We have followed the author guideline of the PLOS ONE and we have corrected the given comments in the revised manuscript accordingly.

2. Data Availability statement

- This manuscript contains the minimal data set to reach the conclusions.

**Reviewer’s comments**

**Reviewer #1:**

1. The study has been written in a comprehensive and scientific language and followed the suitable methodology and statistical testing. Only to revise the conclusion and stick to the aim. Generally, no major flaws.

**Authors’ response to reviewer #1**

- We the authors have tried to revise the conclusion and focused on the aim of the paper.

**Reviewer #2:**

1. The paper needs revision in view of spelling, English grammar.
2. Plagiarism was found which was more than expected
3. Key word, prevalence ‘P’ letter was written in a lower letter
4. Since the topic of this manuscript focuses on Prevalence MDR in East African countries, the study area was not included countries fairly in the study, e.g. Djibouti, Somalia, Eritrea, and the rest and also the included countries not equally or fairly distributed the study area according to table 1 page 8 and line 202 e. g Ethiopia 5 times whereas Rwanda is one time and so on

**Authors’ response to reviewer #2**

1. We have revised the manuscript to correct spelling and English grammar.
2. We have tried our best to minimize and avoid plagiarism in this manuscript.
3. In the key word prevalence the letter ‘P’ is written in capital letter.
4. The study focuses in East Africa countries, but some East Africa countries like Djibouti, Somalia, and Eritrea there is studies concerning MDR-TB among new PTB cases and previously treated PTB cases in such countries. That is why we excluded from the study. However, some countries like Ethiopia more studies and Rwanda with one study concerning MDR-TB among new PTB cases and previously treated PTB cases were included in the study since they fulfill the inclusion criteria.

**Reviewer #3:**

1. The English language in the entire script needs serious attention and is not as per the standards.
2. The data sets for example Range et al which has been included in the analysis has the data collected during 2001 and 2002 period and the study states that the data is from 2007, so this has to be carefully checked and included or excluded in the study.

**Authors’ response to reviewer #3**

1. The English language is revised to maintain the standard of the manuscript as much as possible
2. The data sets for Range et al. used for data analyzed are not during 2001 and 2002 period. It is Range et al. 2012 which is after 2007.

**Reviewer #4**

1. My comments of minor nature are as under:-Language issues are in plenty. For eg. in line 273-276, authors state that their MDR-TB estimates were six times higher as compared to the global average reported by WHO. I am sure the authors do not mean this. Similarly, some of the terms are used loosely like new MDRTB, newly diagnosed MDR TB, previously diagnosed MDR TB, etc. What the authors mean is MDR among newly diagnosed TB patients and MDR among previously treated for TB patients. They should be consistent and careful in using terms. Line 70: cure rate among MDR TB remains below 100%: Need to reframe. There are language issues in line 181-182. Some of the references do not seem to be pertinent; for example, ref 1 &2. Line 86-87: history of TB infection be replaced by TB disease

**Authors’ response to reviewer #4**

- The authors have tried to edit the language issues and we have used the phrases MDR among newly diagnosed TB patients and MDR among previously treated for TB patients. The phrase TB infection is replaced with the phrase TB disease. We think that some phrases like cure rate is appropriate to explain the statement. The word infection is replaced with the word disease.
